# Prediction of influenza virus infection based on deep learning and peripheral blood proteomics: A diagnostic study

**DOI:** 10.1016/j.jare.2025.03.051

**Published:** 2025-03-28

**Authors:** Yumei Zhou, Pengbo Wang, Haiyun Zhang, Taihao Wang, Shuai Han, Xin Ma, Shuang Liang, Minghua Bai, Pengbei Fan, Lei Wang, Ji Wang, Qi Wang

**Affiliations:** a National Institute of TCM Constitution and Preventive Treatment of Disease, Wangqi Academy of Beijing University of Chinese Medicine, Beijing University of Chinese Medicine, Beijing 100029, PR China; b Xinxiang Medical University, Xinxiang, Henan 453003, PR China; c Medical Laboratory Center, Dalian Municipal Women and Children’s Medical Center (Group), Dalian, Liaoning 116033, PR China; d Capital Medical University, Beijing 100069, PR China; e Inner Mongolia Medical University, Hohhot, Inner Mongolia 010070, PR China; f China Railway Construction Corporation, Beijing Tiejian Hospital, Beijing 100039, PR China; g Department of Radiology, The Second Affiliated Hospital to Mudanjiang Medical University, Mudanjiang, Heilongjiang 157000, PR China; h Hubei Shizhen Laboratory, Hubei University of Chinese Medicine, Wuhan, Hubei 430065, PR China

**Keywords:** SAA2, Influenza, COVID-19, Proteomics, Deep learning

## Abstract

**Introduction:**

Influenza viruses cause seasonal epidemics almost every year, and it is difficult to diagnose quickly and accurately. Machine learning and peripheral blood protein omics have brought new ideas to the research of clinical markers.

**Objectives:**

Prediction of key molecular marker of influenza virus infection by the established machine learning model and peripheral blood protein omics.

**Methods:**

This study used the testing data of 850 patients (including influenza, COVID-19 and mixed infections) and 265 healthy individuals, to establish and validate a diagnostic prediction model for influenza infection and verified the potential value of this model in the differential diagnosis of influenza, COVID-19 and healthy people.

**Results:**

The overall analysis showed that there were significant differences in 9 clinical features in the influenza group. Principal component analysis can effectively group samples based on these clinical features. Based on the random forest model and LASSO regression model found that the selected features are clinical indicators that can accurately distinguish influenza patients. We performed proteome sequencing combined with machine learning and found a total of 26 DEPs. Through PPI and WGCNA analysis, we identified several genes related to the proportion of monocytes. We then analyzed the correlation of these factors with immune cell proportions and found that SAA1 and SAA2 were highly correlated with various vital immunocyte. ROC curve analysis shows that SERPINA3 can distinguish influenza, COVID-19, mixed infection and healthy people; SAA1 can distinguish COVID-19, mixed infection and healthy people; SAA2 can distinguish influenza and healthy people. In influenza, high expression of SERPINA3, SAA1, and SAA2 is associated with higher risk. Finally, we used the ELISA method to confirm that SAA2 protein can be used as an auxiliary diagnostic indicator for influenza infection.

**Conclusions:**

Preliminary results showed that SAA2 is an important molecular marker specific to influenza infection.

## Highlights

• Machine learning and proteomics enhance influenza diagnosis.
• Bioinformatics analysis identifies key biomarkers for influenza.
• SAA2 as an auxiliary diagnostic marker for influenza infection.
• Accurate classification based on clinical features and protein biomarkers.

## Graphical abstract

**Overview of the study design.** The participants were divided into four groups: control group (n=265), influenza group (n=225), COVID-19 group (n=358), and mixed group (n=267). Principal component analysis was used to group samples according to clinical characteristics. Selected features were analyzed based on random forest model and LASSO regression model. Proteome sequencing combined with machine learning methods were used for analysis. ROC curve analysis was used to analyze the clinical diagnostic efficacy of inflammatory factors among different groups. Risk prediction analysis was used to analyze the relationship between high expression of some inflammatory factors in COVID-19 cases and higher risk of disease. ELISA was used to detect the levels of different inflammatory factors in plasma of different populations.

**Figure:**
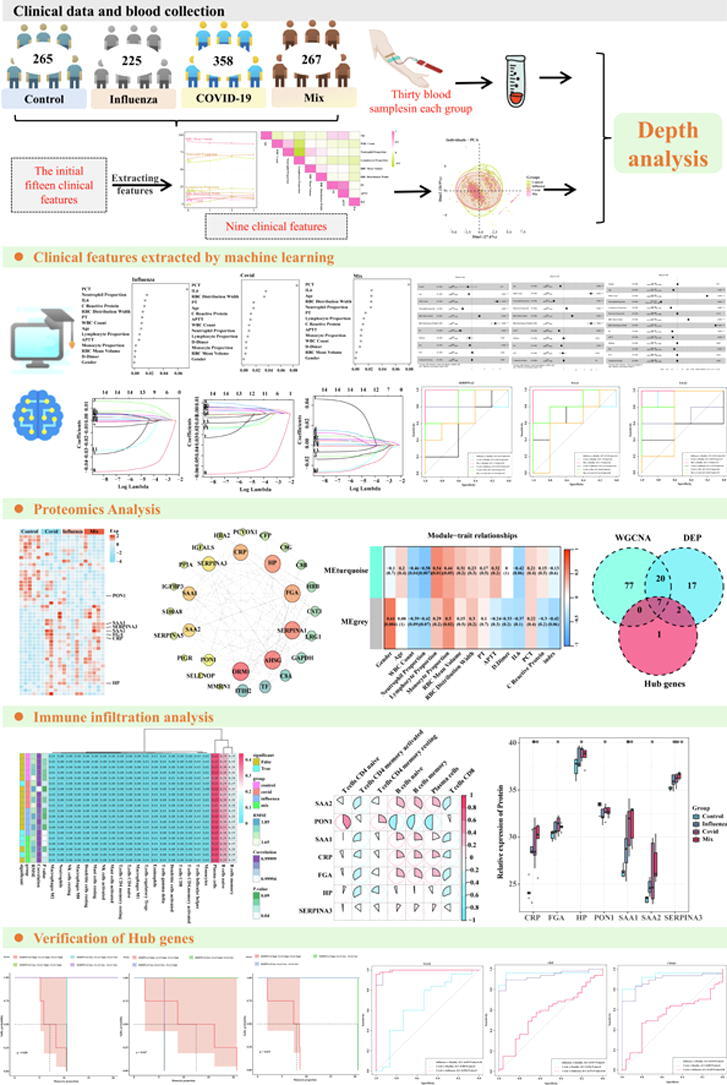

## Background

Influenza viruses cause seasonal epidemics almost every year. They are highly contagious respiratory infections that spread rapidly from person to person through respiratory droplets and direct contact, and can cause severe illness or even life-threatening conditions in some high-risk groups [1]. Accurate and rapid diagnosis of infection is difficult. Nucleic acid amplification tests for influenza viruses can only diagnose whether a person is currently infected with this specific virus, but do not provide information about other diseases or symptoms [2]. Serological tests detect viral antibodies (IgG and IgM) in serum or plasma, which can help assess immune responses, track disease progression, and the duration of immune protection after patients recover from the disease [3]. However, the production of antibodies takes some time (usually 7–14 days) and cannot be used as a rapid diagnostic indicator [4]. Therefore, the identification of molecular markers for disease diagnosis is very important for disease treatment.

Ideally, clinical disease diagnosis is best achieved through non-invasive sample collection, which can be repeatedly collected from patients in a short period of time. For example, biological fluids such as plasma, serum, or urine are the best choices [5]. Plasma and serum are relatively easy to handle and store, making them the most common samples for systemic disease research. This is because blood protein levels reflect the cumulative effects of diverse biological processes across organ systems. Different classes of pathogens trigger specific pattern recognition receptors that are differentially expressed on leukocytes [6]. Blood serves as both a reservoir and a migration zone for these cells, which may be exposed to infectious pathogens, allergens, tumors, transplants, or autoimmune responses [7]. Blood testing is therefore an accessible source of clinically relevant information. Omics (e.g., proteomics) data can provide a comprehensive overview of disease-related cellular processes and provide insights into the complexity of disease [8]. Biological insights can be combined with clinical and social data and applied in clinical settings to improve health outcomes. Because diseases are complex, evolve over time, and exhibit incredible heterogeneity among individuals, omics analysis can provide deep phenotyping of individuals’ trajectories from health to disease, enabling a precision health approach that could lead to earlier and/or more effective interventions [9]. Based on plasma proteomics, Sahin et al. identified a total of 1,147 proteins and 111 significantly altered plasma proteins from 52 COVID-19 samples and constructed a COVID-19-associated network [10]. However, these studies focused primarily on proteomics and clinical features while neglecting deep learning features that could provide more valuable insights. With the accumulation of large amounts of patient records and data, and the rise of the trend toward personalized treatment, the demand for automated, reliable health information processing and analysis is increasing. To meet this demand, deep learning algorithms have gradually become one of the most promising algorithms for multivariate data analysis in recent years due to their excellent prediction performance and ability to capture nonlinear and hierarchical features. Currently, no study has combined proteomic features and deep learning features to analyze influenza virus infection.

This study used the peripheral plasma proteomics and deep learning features of healthy controls, patients with influenza alone, patients with COVID-19 alone, and patients co-infected with influenza and COVID-19 to establish and validate a diagnostic prediction model for influenza infection and verified the potential value of this model in the differential diagnosis of influenza, COVID-19 and healthy people. Preliminary results identified SAA2 as a specific molecular marker for influenza infection.

## Methods

### Clinical data and blood collection

In this study, we retrospectively analyzed the test data of 850 patients (influenza, COVID-19, and mixed infections of the two diseases) and 265 healthy people (control group) who were treated in the Second Affiliated Hospital of Mudanjiang Medical College from January to June 2023. Thirty blood samples each from healthy people, COVID-19 and influenza infected patients were collected. This study was conducted in strict accordance with the Declaration of Helsinki and was approved by the Medical Ethics Review Committee of the Second Affiliated Hospital of Mudanjiang Medical College (No. 202328). All participants gave their consent to participate in the study.

### Label-free protein profiling detection

Protein quantification was performed using the Bradford protein quantification kit (purchased from Shanghai Biotechnology Company). Trypsin (purchased from Solebac (Beijing)) was added to each protein sample and incubated at 37 °C for 4 h. CaCl_2_ was added to each sample and digested overnight. Formic acid was added, centrifuged at 12,000 × g for 5 min at room temperature, and the resulting supernatant was loaded onto a C18 desalting column. LC-MS/MS analysis was performed using an EASY-nLC TM 1200 UHPLC and Q Exactive TM HF-X mass spectrometry system, both purchased from ThermoFisher (USA), operated in data-dependent acquisition (DDA) mode. The differences in protein abundance between groups were examined using a *t*-test, and proteins with a fold change > 1.2 and *p* < 0.05 were considered differentially expressed proteins.

### Data processing

Missing value processing: Continuous variables were filled using linear imputation, and categorial variables were filled using mode. All variables had no missing values, and all continuous variables were normalized by logarithmtransformation.

### Clinical features extract

Based on the collected clinical information, we performed baseline data analysis and extracted 9 clinical features that showed significant intergroup differences out of the initial 15 clinical features. Using the “lm” function in R, we constructed a linear regression formula based on the groups: *Index = 0.006 × Age − 0.045 × WBC Count + 0.01 × Lymphocyte Proportion + 0.025 × Neutrophil Proportion − 0.017 × RBC Mean Volume − 0.059 × RBC Distribution Width + 0.008 × PT + 0.007 × APTT + 0.005 × IL6 + 1.6*.

The correlation between clinical features was analyzed using the chi-square test and visualized with a heat map. Principal component analysis (PCA) was performed using the “prcomp” function from the “stats” R package based on the 9 clinical features. We utilized the R package “randomForest” (Version 4.7) to construct a random forest model for ranking the importance of these clinical features and evaluating their performance as indicators. Specifically, the number of decision trees (such as n tree ≥ 500), the maximum depth and other parameters are set, and the Gini index decline or replacement importance is used to evaluate the feature contribution. Finally, the least absolute shrinkage and selection operator (LASSO) regression was applied for feature selection. Non-zero coefficients were considered valuable predictors in each feature group. In brief, LASSO Cox regression analysis was performed using the R package “glmnet” (Version 4.1) to narrow down the candidate genes and develop the prognostic model. The penalty parameter (λ) was determined based on the minimum parameters. Risk scores were calculated using the “*Index*”, which was derived from data in the training set.

### PCA and DEP analysis

Proteomics data were processed by R packages “DEP” (Version 1.26.0). Proteomic data were then normalised using “normalize_vsn” function. PCA was used to conduct clustering analysis of the samples by “plot_pca” function. “add_rejections” function was applied to calculate the fold-change values of proteins. In this study, a fold change > 1 or < 0.5, and false-discovery rate adjusted *P* value (*t* test) < 0.05 were set for differential expression proteins filtration.

### Functional enrichment and PPI analysis

Gene ontology (GO) enrichment analysis of DEPs was implemented in the GOseq R packages based on Wallenius non-central hypergeometric distribution (https://geneontology.org/). The DEPs were analyzed using the Kyoto Encyclopedia of Genes and Genomes (KEGG) database (https://www.genome.jp/kegg/pathway.html). Protein-protein interaction network was conducted in STRING (https://string-db. org/).

### Construction of co-expression network

Weighted gene co-expression network analysis (WGCNA) was utilized to identify gene modules with similar expression patterns and analyze the correlation between monocyte proportion and gene modules. The scale independence and average connectivity of the networks were tested with different power values (from 1 to 20). The appropriate power value was determined when the independent scale was greater than 0.85 and the connectivity was high. Then, the similarity matrix was transformed into a topological matrix with the topological overlap measure (TOM) describing the correlation between genes. The genes were clustered by using 1-TOM as the distance. A dynamic hybrid cutting method was used to establish a hierarchical clustering tree to identify co-expressed gene modules. Each leaf of the tree represents a gene, and genes with similar expression data aggregate to form a branch of the tree and each branch represents a gene module. The correlation between module feature genes and monocyte proportion was calculated using Pearson correlation analysis, and the genes in the modules most relevant to the monocyte proportion in the four group were intersected.

### Immune infiltration analysis

Proteomics data was transformed into the total abundance of immune cells by utilizing the Cell-type Identification by Estimating Relative Subsets of protein (CIBERSORT) analysis with the “CIBERSORT” (Version 0.1.0) R package.

### Survival analysis

Survival analysis was performed by R package survival (Version 3.5). Hazard ratio (HR) was calculated by Cox proportional hazards model and 95 % CI was reported, and Kaplan–Meier survival curve was modeled by “survfit” function. The two-sided long-rank test was used to compare Kaplan–Meier survival curves.

### ELISA

The three proteins studied, vWF, CD163 and SAA2 kits, were purchased from Zeye Biotechnology Company (China). All plasma samples were freshly collected clinical non-anticoagulated blood, centrifuged at 4000r/min for 10 min. According to the kit instructions, add 50 µL of antigen-containing serum and standard into the small wells, followed by adding 50 µL of reaction reagent containing primary antibody, cover the plate with adhesive plastic and incubate at 37 °C for 2 h. Wash the wells 3 times with 250 µL wash buffer (0.05 % Tween 20 in PBS). Horseradish peroxidase-conjugated secondary antibody was added and incubated for 30 min at 37 °C. Wash the plate five times with 250 µL wash buffer, then add 100 µL TMB reagent to each well for color development. After 10 min, add 100 µL of stop buffer to the wells and record the absorbance value at 450 nm.

### Statistical analysis

The normal distribution of the data was assessed by the Shapiro-Wilk normality test using GraphPad Prism 8.0 (USA) or SPSS Statistics version 17.0 (IBM, USA), and the mean is expressed as mean ± standard error. If the data were normally distributed, the differences between the means were assessed by one-way analysis of variance and Tukey’s multiple comparison test. If the data were not normally distributed, scores were compared using the nonparametric Kruskal-Wallis test. When *p* < 0.05, the difference between groups was considered statistically significant.

## Results

### Clinical data analysis

A total of 1115 samples were collected and divided into four groups: Control (n = 265), Influenza (n = 225), COVID-19 (n = 358), and Mix (n = 267). Significant differences were observed among the groups in terms of Age, WBC Count, Neutrophil Proportion, Lymphocyte Proportion, RBC Mean Volume, RBC Distribution Width, PT, APTT, and IL6 levels (**Table S1**).

### Analysis and importance Ranking of clinical sample features

**Fig. 1:**
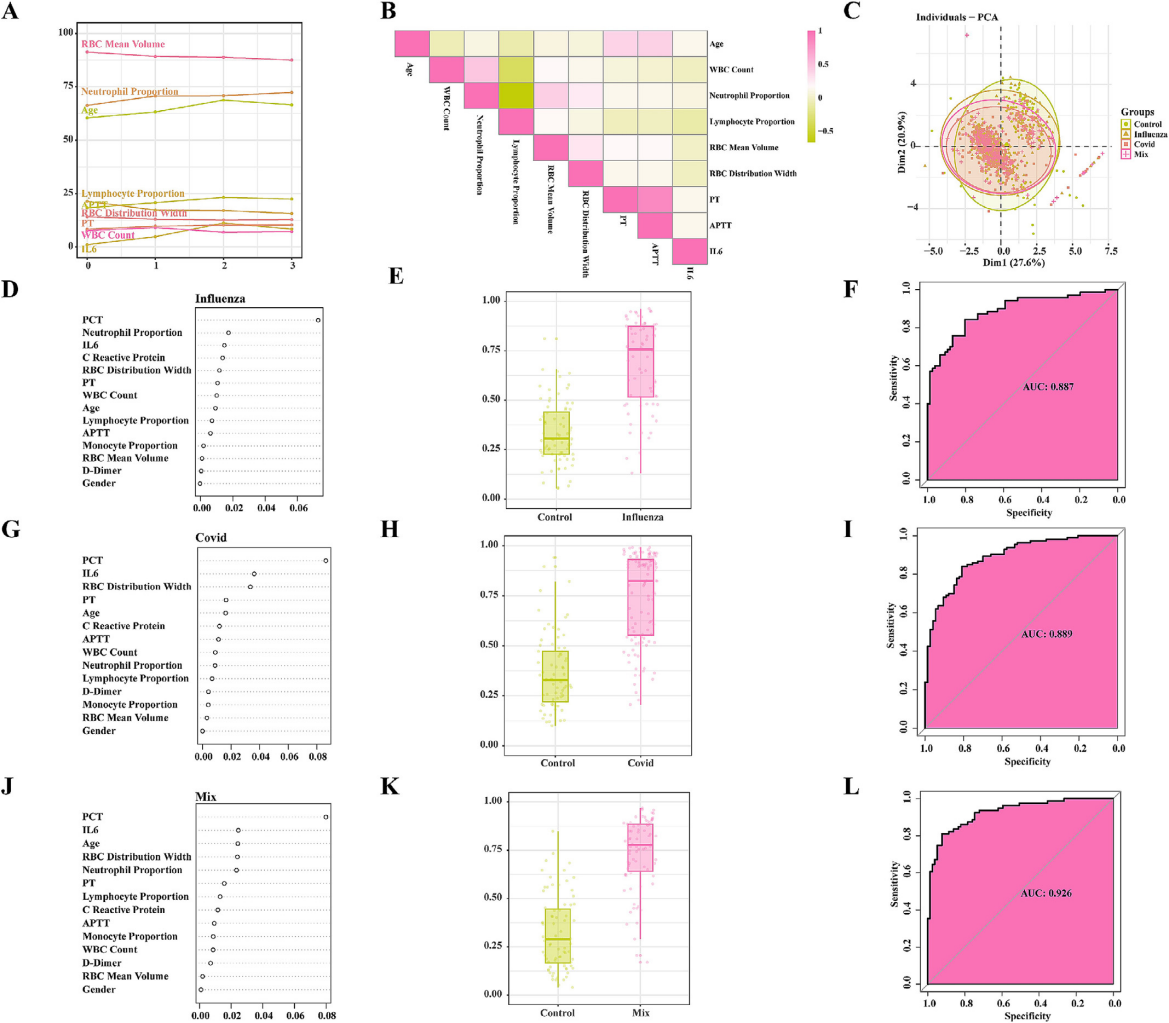
**Analysis and importance ranking of clinical sample features.** A. Trends in Clinical Sample Features Across Groups; B. Correlation Analysis of Clinical Sample Features; C. PCA Dimensionality Reduction of Clinical Samples; D. Feature Ranking of Influenza Random Forest Model; E. Evaluation of Test Set Predictive Performance of Influenza Random Forest Model; F. Performance Evaluation of Influenza Random Forest Model; G. Feature Ranking of COVID-19 Random Forest Model; H. Evaluation of Test Set Predictive Performance of COVID-19 Random Forest Model; I. Performance Evaluation of COVID-19 Random Forest Model; J. Feature Ranking of Mix Random Forest Model; K. Evaluation of Test Set Predictive Performance of Mix Random Forest Model; L. Performance Evaluation of Mix Random Forest Model.

To further screen the clinical sample features for machine learning model construction, we analyzed the changing trends of significant clinical sample features among different groups. The results revealed that Age, APTT, PT, IL6, and Neutrophil Proportion exhibited similar changing trends among different groups, while RBC Mean Volume, Lymphocyte Proportion, RBC Distribution Width, and WBC Count also showed similar changing trends (Fig. 1A). Furthermore, the correlation analysis among the clinical features demonstrated that Age was positively correlated with PT and APTT, WBC Count was positively correlated with Neutrophil Proportion, Neutrophil Proportion was positively correlated with RBC Mean Volume and RBC Distribution Width, and RBC Mean Volume was positively correlated with RBC Distribution Width. Additionally, Neutrophil Proportion showed a negative correlation with Lymphocyte Proportion (Fig. 1B).

PCA shows that the horizontal and vertical coordinates (Dim 1 and Dim 2) explain 27.6 % and 20.9 % of the total variation respectively, and the cumulative contribution rate is 48.5 %, which indicates that it can effectively capture the main variation trend of samples data (Fig. 1C). Subsequently, linear fitting was performed on these nine clinical features based on the grouping. This formula was used to construct a new composite index that incorporates the variations of these clinical features for outcome classification.

In the random forest model, the clinical features' importance in the Influenza group, from highest to lowest, are as follows: PCT, Neutrophil Proportion, IL6, etc. (Fig. 1D). Among them, RBC Distribution Width, WBC Count, and RBC Mean Volume may serve as risk factors for Influenza (Fig. S1A). The model constructed using these features showed excellent predictive performance on the test set (Fig. 1E) and had good diagnostic efficacy (AUC = 0.887) (Fig. 1F). For the COVID-19 group, the importance of clinical features from high to low was: PCT, IL6, RBC distribution width, etc. (Fig. 1G). Among them, RBC distribution width, WBC count, monocyte ratio and RBC average volume may be risk factors for COVID-19 (Fig. S1B). The model constructed using these features showed excellent predictive performance on the test set (Fig. 1H) and had high clinical diagnostic value (AUC = 0.889) (Fig. 1I). For the Mix group, the importance of clinical features from high to low was: PCT, IL6, age, etc. (Fig. 1J). Among them, RBC distribution width, WBC count and RBC average volume can be regarded as risk factors for COVID-19 (Fig. S1C). The model constructed using these features showed excellent predictive performance on the test set (Fig. 1K) and good overall diagnostic performance (AUC = 0.926) (Fig. 1L).

### Selection of important feature variables using LASSO Regression

**Fig. 2:**
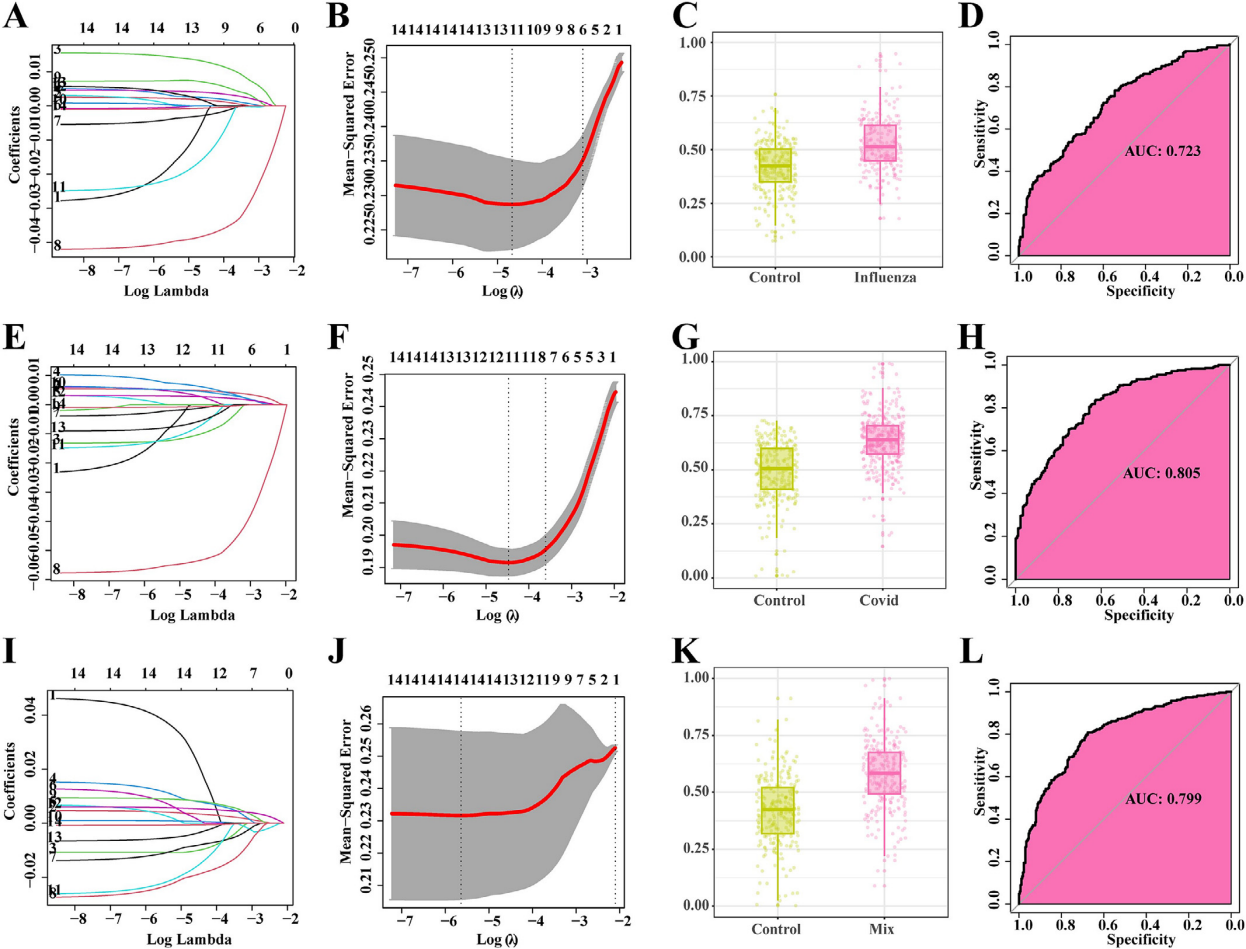
**selection of important feature variables using LASSO Regression.** A. LASSO Regression Coefficients of Influenza Clinical Features, with Different Colored Lines Representing the Coefficients of Each Feature; B. Selection of Optimization Parameter (lambda) in Influenza LASSO Regression Model; C. Prediction in Influenza Test Set; D. Performance Evaluation of Influenza LASSO Regression Model; E. LASSO Regression Coefficients of COVID-19 Clinical Features, with Different Colored Lines Representing the Coefficients of Each Feature; F. Selection of Optimization Parameter (lambda) in COVID-19 LASSO Regression Model; G. Prediction in COVID-19 Test Set; H. Performance Evaluation of COVID-19 LASSO Regression Model; I. LASSO Regression Coefficients of Mix Clinical Features, with Different Colored Lines Representing the Coefficients of Each Feature; J. Selection of Optimization Parameter (lambda) in Mix LASSO Regression Model; K. Prediction in Mix Test Set; L. Performance Evaluation of Mix LASSO Regression Model.

The selection process of clinical features for Influenza is illustrated in Fig. 2A and Fig. 2B. Using these selected clinical features, we constructed a LASSO regression model and applied it to predict the test dataset. The results demonstrated that the model effectively differentiated between healthy individuals and Influenza patients (Fig. 2C). Moreover, the model exhibited favorable performance with an AUC of 0.723 (Fig. 2D). Similarly, the selection process of clinical features for COVID-19 is depicted in Fig. 2E and Fig. 2F. By constructing a LASSO regression model using these selected features, we achieved accurate discrimination between healthy individuals and COVID-19 patients (Fig. 2G). The model also demonstrated high performance with an AUC of 0.805 (Fig. 2H). For the Mix group, the selection process of clinical features is presented in Fig. 2I and Fig. 2J. The constructed LASSO regression model effectively distinguished between healthy individuals and Mix patients (Fig. 2K). The model exhibited excellent performance with an AUC of 0.799 (Fig. 2L). Additionally, in the Influenza, COVID-19, and Mix groups, the coefficients of different feature values are summarized in Table S2.

### Proteomics analysis

**Fig. 3:**
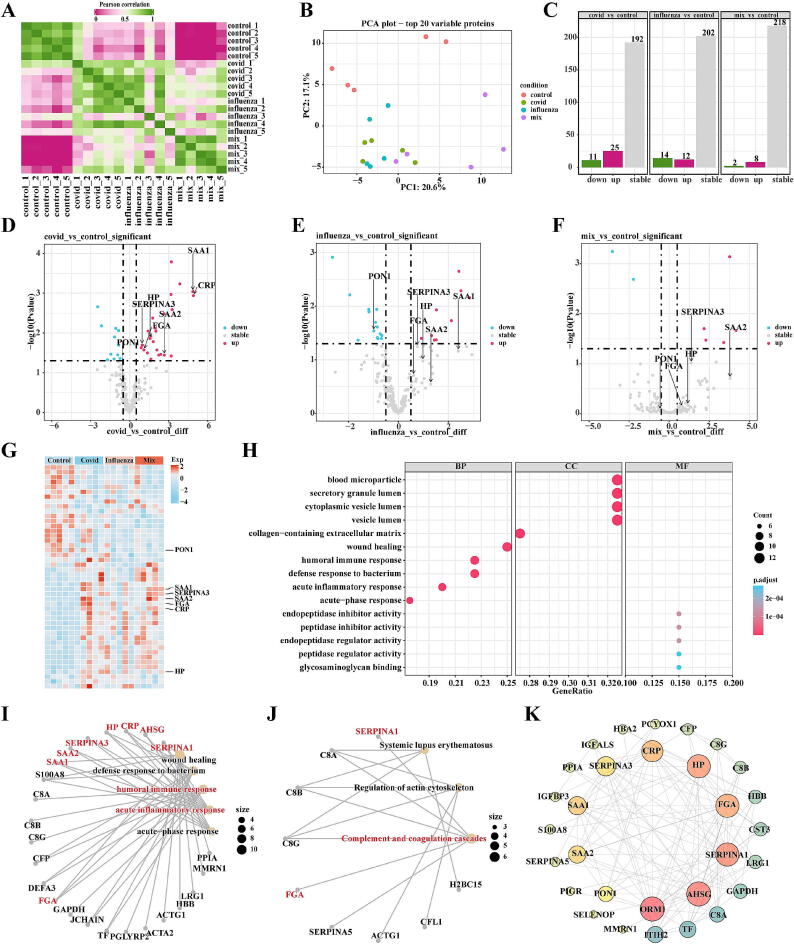
**proteomic analysis.** A. Similarity analysis between samples; B. PCA dimensionality reduction of samples; C. Differential protein analysis; D. Volcano plot of differential proteins between COVID-19 and Control; E. Volcano plot of differential proteins between Influenza and Control; F. Volcano plot of differential proteins between Mix and Control; G. Heatmap of differential protein expression; H. GO enrichment analysis of differential proteins; I. Network plot of GO enrichment analysis for differential proteins; J. Network plot of KEGG enrichment analysis for differential proteins; K. Protein-Protein Interaction (PPI) network analysis of differential proteins.

In order to identify biomarkers between COVID-19, Influenza, and Mix, we conducted proteomic sequencing. The similarity between samples is shown in Fig. 3A, where there is a significant difference between the control and COVID-19, Influenza, and Mix groups. However, COVID-19, Influenza, and Mix show a high degree of similarity, indicating a potential consistency in the biological mechanisms underlying the onset of COVID-19, Influenza, and Mix. PCA results demonstrate that COVID-19 has a smaller difference compared to Influenza, but a larger difference compared to Mix. Differential expression analysis was performed using the criteria of logFC > |1| and *p* < 0.05 to identify differentially expressed proteins (DEPs).

We found a total of 36 DEPs (Table S3, Fig. S2A) in COVID-19 *vs* Control, with 11 downregulated proteins and 25 upregulated proteins. In Influenza *vs* Control, there were 26 DEPs (Table S4, Fig. S2B), including 14 downregulated proteins and 12 upregulated proteins. In Mix *vs* Control, there were 10 DEPs (Table S5, Fig. S2C), with 2 downregulated proteins and 8 upregulated proteins (Fig. 3C-F). The expression levels of these differential proteins are shown in Fig. 3G. To further understand the functions of these DEPs, we performed enrichment analysis. The results revealed that these differentially expressed genes are mainly enriched in biological processes such as acute-phase response, acute inflammatory response, defense response to bacterium and human immune response. They are also enriched in the vesicle lumen component and possess peptidase regulatory activity (Fig. 3H). The genes involved in the enriched biological processes are shown in Fig. 3I, while the DEPs in the enriched signaling pathways are shown in Fig. 3J.

To further identify potential biomarkers, we conducted PPI network analysis using the DEPs and calculated hub genes using cytohubba. The hub genes identified were ORM1, AHSG, SERPINA1, FGA, HP, CRP, SERPINA3, SAA1, SAA2, and PON1, as shown in Fig. 3K.

### WGCNA analysis

**Fig. 4:**
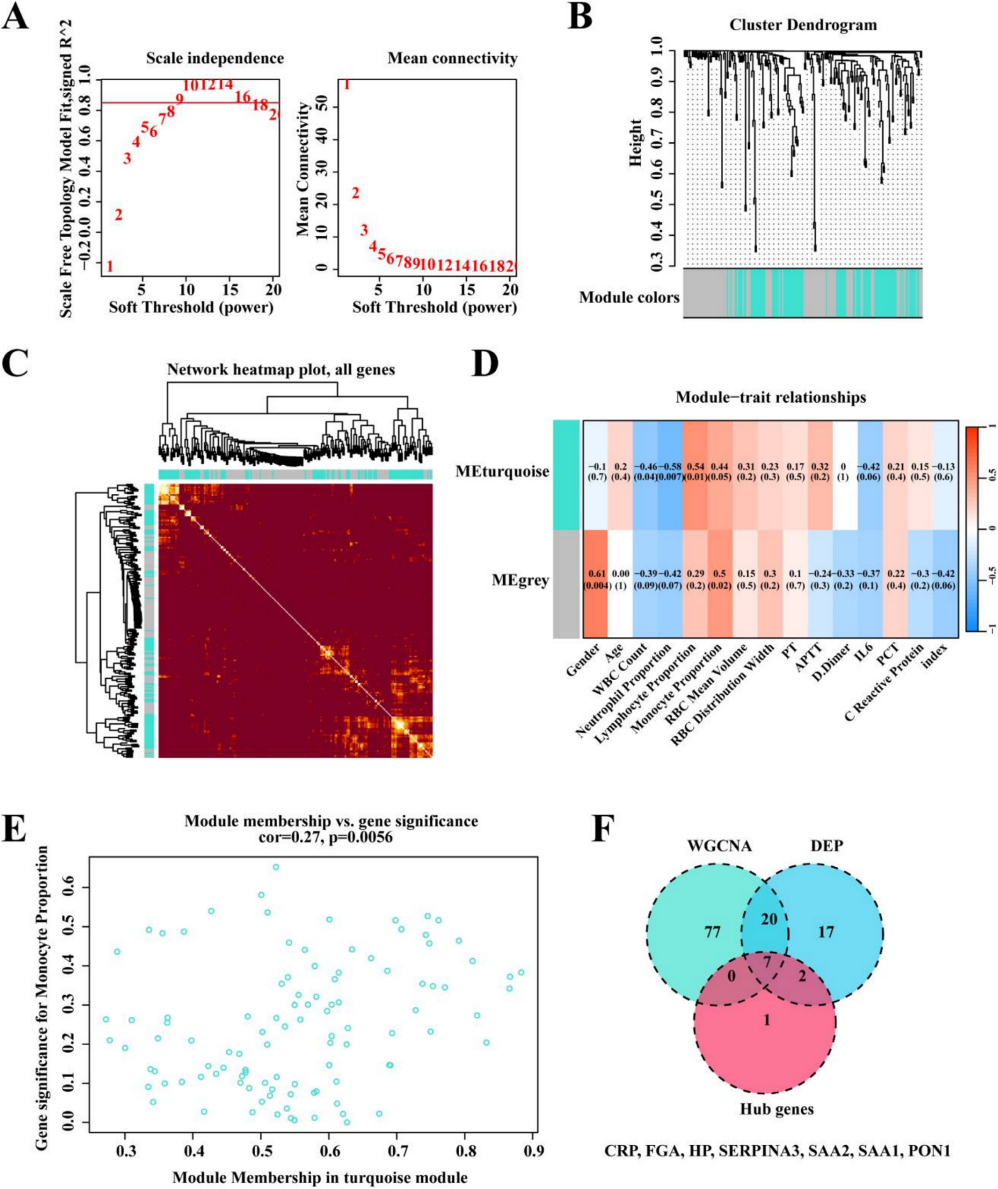
**weighted correlation network analysis.** A. WGCNA soft thresholding selection process; B. Scale-free network; C. Topological overlap heatmap; D. Module-sample trait correlation heatmap; E. Correlation between genes in the turquoise module of the Lymphocyte proportion phenotype; F. Proteins in the turquoise module of the Lymphocyte proportion phenotype, intersecting with differentially expressed proteins and hub genes.

To identify biomarkers for diagnosing COVID-19, Influenza, and Mix, we conducted WGCNA analysis to identify differentially expressed proteins associated with phenotypes. A soft threshold of 9 was chosen to construct a scale-free network (Fig. 4A). Two modules were obtained (Fig. 4B), and a topological overlap heatmap was generated using all proteins (Fig. 4C). Based on the WGCNA results, we selected genes in the “turquoise” module from the Lymphocyte proportion phenotype for further investigation (Fig. 4D). The expression correlation of genes in this module is shown in Fig. 4E. Subsequently, we selected proteins from this module that intersected with differentially expressed proteins and hub genes. We identified CRP, FGA, HP, SERPINA3, SAA2, SAA1, and PON1 as potential biomarkers (Fig. 4F).

### Immunoinfiltration analysis

**Fig. 5:**
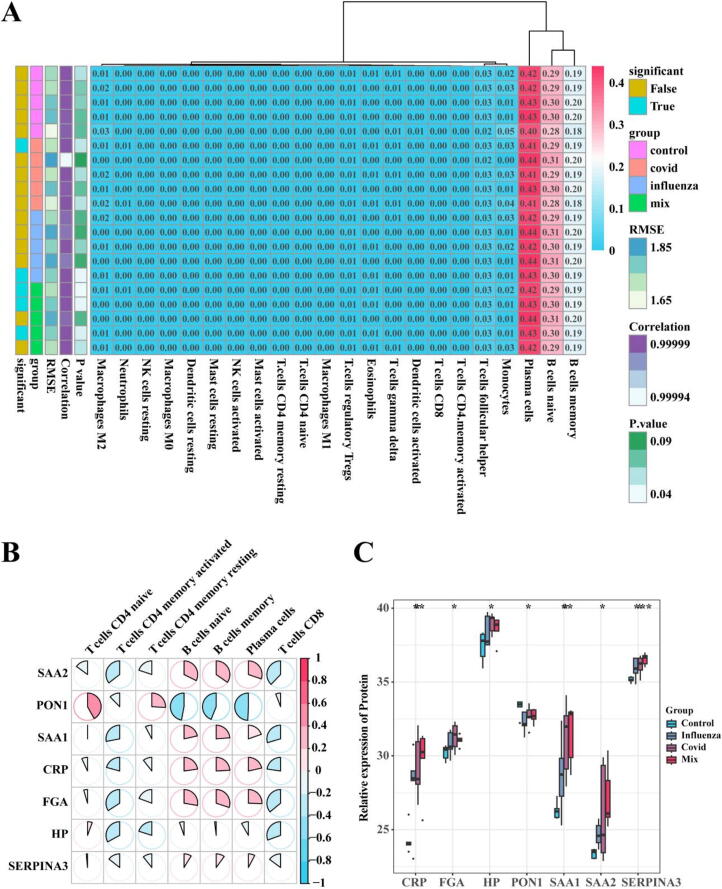
**immunoinfiltration analysis.** A. Proportions of Immune Cells in Different Samples; B. Correlation between CRP, FGA, HP, SERPINA3, SAA2, SAA1, PON1, and Immune Cell Proportions; C. Expression Patterns of CRP, FGA, HP, SERPINA3, SAA2, SAA1, PON1.

To further confirm the suitability of HP, SERPINA3, SAA1, and SAA2 as biomarkers, we conducted an analysis of immune cell proportions in different samples. The results showed that the highest proportions of immune cells in various samples were Plasma cells, B cells memory, and naïve B cells (Fig. 5A). We then analyzed the correlation between CRP, FGA, HP, SERPINA3, SAA2, SAA1, PON1, and the proportions of immune cells. The results indicated that SAA1 and SAA2 had a higher correlation with the proportions of Plasma cells, B cells memory, and naïve B cells (Fig. 5B). The expression patterns of CRP, FGA, HP, SERPINA3, SAA2, SAA1, and PON1 are shown in Fig. 5C. Based on the expression results, SAA1, SAA2, and SERPINA3 may be more suitable as biomarkers.

### Biomarker identification

Validation of SERPINA3, SAA1, and SAA2 as biomarkers for influenza, COVID-19, and Mix. To determine whether SERPINA3, SAA1, and SAA2 can serve as biomarkers for influenza, COVID-19, and Mix, we classified the three genes. The ROC curve showed that SERPINA3 could only distinguish between influenza, COVID-19, Mix, and health (Fig. S3A); SAA1 could only distinguish between COVID-19, Mix, and health (Fig. S3B); and SAA2 could only distinguish between influenza and health (Fig. S3C). These results suggest that multiple biomarkers are needed to distinguish between health, influenza, COVID-19, and Mix.

**Fig. 6:**
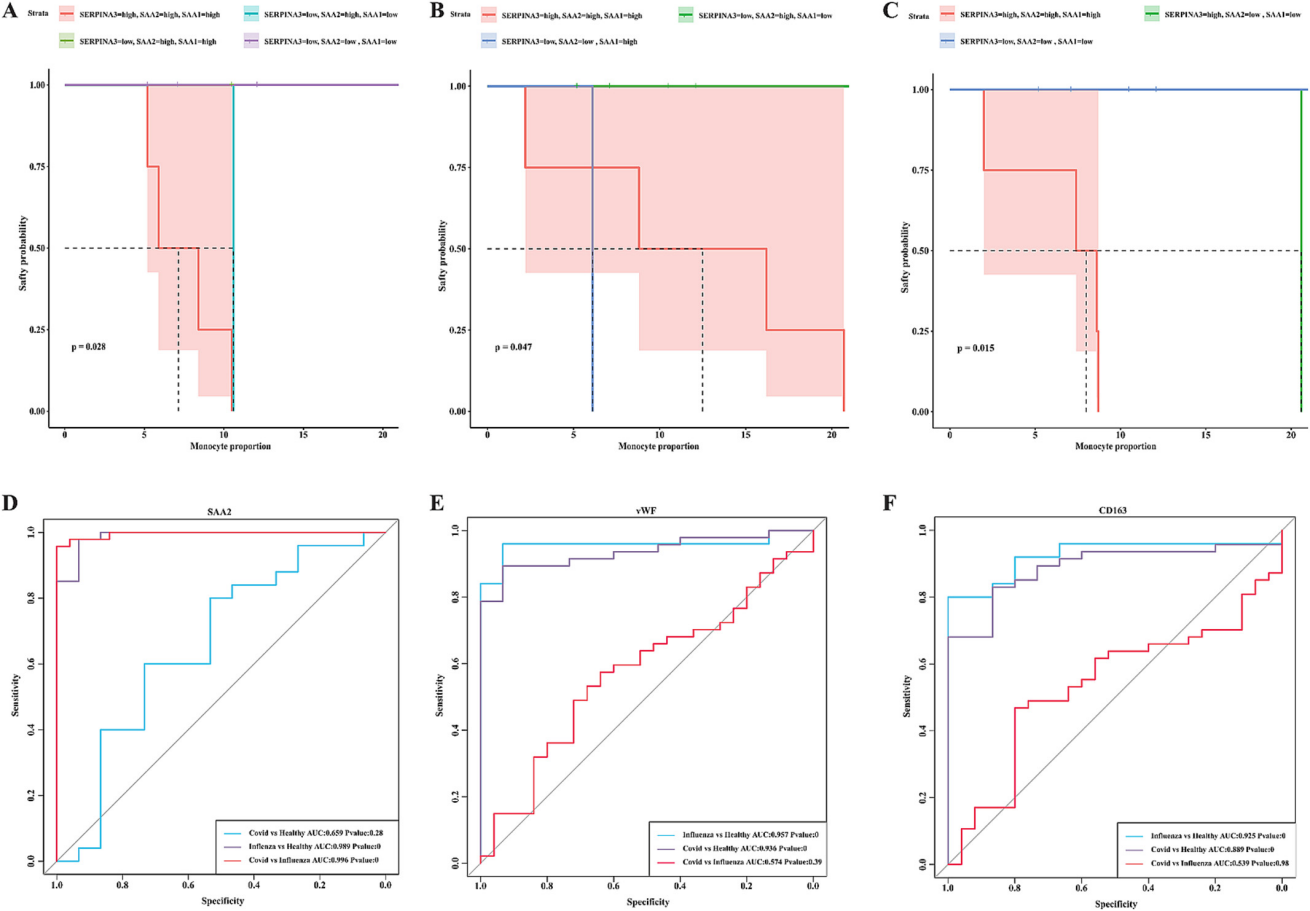
**biomarker identification.** A. Risk assessment of SERPINA3, SAA1, and SAA2 genes in Influenza; B. Risk assessment of SERPINA3, SAA1, and SAA2 genes in COVID-19; C. Risk assessment of SERPINA3, SAA1, and SAA2 genes in Mix; D. ROC curve for **SAA2** (Covid *vs* Healthy, AUC = 0.659; Influenza *vs* Healthy, AUC = 0.989; Covid *vs* Influenza, AUC = 0.996); E. ROC curve for **vWF** (Covid *vs* Healthy, AUC = 0.957; Influenza *vs* Healthy, AUC = 0.936; Covid *vs* Influenza, AUC = 0.574); F. ROC curve for **CD163** (Covid *vs* Healthy, AUC = 0.925; Influenza *vs* Healthy, AUC = 0.889; Covid *vs* Influenza, AUC = 0.539).

Next, we performed a risk prediction analysis. The results showed that in influenza, high expression of SERPINA3, SAA1, and SAA2 was associated with a higher risk (Fig. 6A). In COVID-19, when the proportion of monocytes was less than 6 %, high expression of SERPINA3, SAA1, and SAA2 indicated a higher risk; conversely, when the proportion of monocytes was greater than 6 %, low expression of SERPINA3 and SAA2 and high expression of SAA1 indicated a higher risk (Fig. 6B); in Mix, high expression of SERPINA3, SAA1, and SAA2 indicated a higher risk (Fig. 6C). Based on the analysis of the proteome and combined with the original data, three possible plasma proteins were selected for further detection of their plasma content. ELISA results showed that SAA2 could clearly distinguish influenza virus from COVID-19 infection and healthy people (Fig. 6D-F), suggesting that it has the potential to be used as a biomarker.

## Discussion

Increases in global molecular diagnostic capabilities have significantly improved our understanding of respiratory virus transmission patterns [11]. Simple routine clinical detection indicators are not sufficient as rapid and reliable clinical markers for the diagnosis of influenza virus infection. In this study, we collected clinical diagnostic data from the peripheral blood of influenza patients using healthy individuals and COVID-19 patients as controls. Subsequently, their peripheral plasma was examined using a proteomic approach and data analysis was performed using advanced machine learning models. Ultimately, we screened potential plasma markers that could be used to diagnose influenza virus infection. It was finally determined that SAA2 protein can be used as a valuable auxiliary diagnostic indicator for influenza virus infection.

In our influenza group, we observed a significant increase in white blood cell count compared to healthy individuals, and the increase was higher than in the COVID-19 control group. However, changes in the proportion of neutrophils and lymphocytes were similar to those in the COVID-19 group. Studies have found that compared with healthy controls, influenza virus-infected patients have significantly increased levels of white blood cells and neutrophils [12], [13]. For COVID-19, Atik et al. found that white blood cell levels in COVID-19 patients were significantly higher than those in controls, with an increased proportion of neutrophils and a decrease in lymphocytes [14]. This finding is consistent with our findings. Neutrophils, as innate immune cells, are the first responders of the inflammatory process and play a key role in the defense against influenza [15]. In addition, a retrospective study showed that there were differences in white blood cell counts between severe and non-severe COVID-19 patients; both groups had elevated white blood cell counts, but the increase in the severe group was significantly higher than that in the severe group, and neutrophils were responsible for the increase. main factors. In addition, another study showed that critically ill patients had a lower proportion of lymphocytes, suggesting that lower lymphocyte counts are associated with poor prognosis [16]. Blood is a “sentinel” for many diseases and an important indicator of infectious diseases [17]. Routine blood tests are widely used for diagnosis and treatment, but their results often need to be interpreted in conjunction with other clinical indicators. Routine blood tests alone often exhibit relatively low sensitivity and specificity when used as diagnostic markers.

Influenza viruses and COVID-19 are two important respiratory viruses that can cause severe respiratory diseases in humans and animals after infection [18]. Influenced by the previous global epidemic of COVID-19, we first observed clinical indicators of COVID-19. Typically, in severe cases of COVID-19, elevated interleukin-6 (IL-6) levels are common. Zhou et al. reported that in severe COVID-19 patients, Th1 cells can induce CD14 ^+^ CD16 ^+^ monocytes to produce IL-6, exacerbating cytokine storm-related inflammation [19]. Disease exacerbation is positively correlated with increased IL-6 levels [20]. Chen et al also found that serum SARS-CoV-2 viral load in severe patients was closely related to IL-6 levels, and high IL-6 levels may lead to neutrophilia and lymphopenia [21]. Our study found that IL-6 levels were significantly increased after COVID-19 infection, but there were no significant changes in influenza patients. Subsequently, it was discovered incidentally that procalcitonin (PCT) levels were significantly elevated following influenza virus infection. Our study found significant differences in peripheral blood PCT levels between influenza and COVID-19.

Influenza is a contagious respiratory disease caused by influenza viruses, whose genetic material is single-stranded negative-sense RNA [22]. In contrast, COVID-19 is caused by an enveloped, single-stranded, positive-sense RNA virus [23]. Innate immunity represents the host's natural defense against pathogens, including viruses, bacteria, and fungi. Specific components of these pathogens, such as nucleic acids and proteins, are known as pathogen-associated molecular patterns (PAMPs). These PAMPs are recognized by pattern recognition receptors (PRRs) on host cells, thereby initiating the innate immune response [24]. Epithelial cells are the primary targets of respiratory viral infections. Following viral infection, these cells secrete proinflammatory mediators and recruit and activate innate immune cells [25]. Various cell types participate in the innate immune response, including polymorphonuclear cells, cytotoxic lymphocytes (particularly natural killer cells), and mononuclear phagocytes [26]. Tissue-resident macrophages and dendritic cells are the first activated innate immune cells. They secrete type I interferons and recruit chemokines to the site of infection, mediate inflammation and promote leukocytes and lymphocytes such as eosinophils. granulocytes, neutrophils, monocytes, and NK cells), thus playing a role in virus clearance and stimulating adaptive immune responses [27]. Activation of the immune system can lead to mild to moderate respiratory illness, characterized by symptoms such as fever and cough. However, elevated cytokine levels can trigger a cytokine storm, which is significantly affected by both innate and adaptive immune cells [28]. It is precisely because the immune responses activated by different pathogens are significantly different, and the immune system produces a variety of inflammatory mediators, resulting in different diagnostic indicators for infection by different pathogens, which brings application value to clinical pathogen diagnosis [29]. Interestingly, PCT levels are particularly elevated in bacterial infections and are considered a marker of bacterial infection [30], [31]. In contrast to this study, Patel et al. found no significant association between PCT levels and site of infection or type of infecting pathogen [32]. This suggests that the physiological mechanism for monitoring PCT values in these viral infections remains unclear and its use as a clinical diagnostic marker remains to be discussed.

Blood is an important component of the immune system and is the main carrier of immune cells throughout the body. Inflammatory factors produced by various organs are released into the blood [33]. This study confirmed that several inflammation-related proteins were significantly increased in patients infected with the influenza virus. Notably, SAA2 emerged as an important biomarker of influenza infection compared with healthy individuals and COVID-19 infected individuals. SAA family members act as acute-phase reactants and chemokines [34]. Of the four known SAA isoforms, SAA1 and SAA2 are highly expressed in the liver of mammals, including humans, in response to inflammatory stimuli, leading to a significant increase in their circulating levels [35]. Because SAA1 and SAA2 are expressed in both liver and extrahepatic tissues in humans, accurately distinguishing their functions in these different sites can be challenging [36]. SAA interacts with formyl peptide-like receptors 1 and 2 (FPLR1 and FPLR2) on human monocytes, neutrophils, and endothelial cells, thereby promoting chemotaxis and promoting immune responses [37]. Recent studies have emphasized that SAA protein, as an acute phase reactant, can amplify the pro-inflammatory response and recruit immune cells during influenza virus infection, and enhance the response of some neutrophils to influenza A virus [38]. This is consistent with the results of WGCNA/PPI, which links SAA1/2 with immune cell infiltration. Our study found that SAA2 is also derived from monocytes in influenza patients. Compared to viral RNA detection (e.g., RT-PCR), which requires high viral load for sensitivity, SAA2 levels rise early in infection due to its role in the acute-phase response. We emphasize that SAA2 is not a standalone diagnostic tool but a supplementary indicator that enhances stratification of disease severity (e.g., differentiating mild *vs*. severe cases) when combined with clinical scores or viral load data.

The mechanisms of serum amyloid A (SAA) expression and secretion vary depending on the type of stimulus. In acute inflammatory responses, such as those seen in sepsis [39], [40], COVID-19 [41], or tissue trauma [42], systemic SAA levels can increase up to 1,000-fold above baseline levels. During infection, most systemic SAA originates primarily from the liver [43], although extrahepatic sources may also play a role after tissue trauma [44]. This makes SAA a valuable clinical diagnostic marker. In mice, expression of SAA1 and SAA2 genes in hepatocytes is regulated by proinflammatory cytokines, including IL-1β, IL-6, and TNF-α [45], [46]. Neutrophils are one of the main target cells of SAA and play an important role in mediating inflammation [47]. Murdoch et al. Used a zebrafish model to demonstrate that SAA promotes neutrophil recruitment to peripheral lesions while limiting clearance of pathogenic bacteria [48]. Studies have confirmed that SAA is significantly up-regulated in patients with influenza virus infection, effectively recruiting neutrophils to the infection site to fight against viral threats [49], [50], [51]. In addition, CRP is currently used clinically as a diagnostic marker for infectious diseases, but CRP increases slightly during viral infection and significantly during bacterial infection [52]. PCT is a precursor of the hormone calcitonin, which is synthesized by parafollicular cells (C cells) of the thyroid gland. It is generally believed that serum PCT levels increase during bacterial infection. During severe bacterial infection (including sepsis), serum PCT levels remain unchanged and do not change significantly during viral infection [53]. Our study found that SAA2 increases significantly during influenza. However, these studies did not clarify which subtype of SAA plays a specific role in influenza infection. Fortunately, there are ready-made SAA2 kits for use in clinical diagnosis, which brings opportunities for our next research.

Deep learning (DL) facilitates the development of computational models with multiple processing layers that can learn representations of data at different levels of abstraction. These models are based on artificial neural networks designed to mimic human brain function [54]. The application of deep learning has grown significantly in recent years, achieving promising results in a range of fields, particularly in image processing, medical image analysis, data analysis, and bioinformatics [55]. In the field of proteomics, deep learning has proven to be very effective in predicting various peptide and protein properties. Notably, DL can bypass traditional peptide and protein identification processes by utilizing data independent acquisition (DIA) data plots as images for convolutional neural network (CNN) classification [56]. This approach can classify samples as diseased or healthy directly from the raw data, rather than relying on identified proteins or peptides. For example, a recent study by Petrovsky et al. One- and three-dimensional CNNs were employed to differentiate renal, ovarian, and prostate cancer patients by analyzing metabolomic datasets [57]. Similarly, Inglese et al. applied deep learning methods to mass spectrometry (MS)-based 3D desorption electrospray ionization (DESI) imaging data to predict heterogeneity in cancer metabolic phenotypes [58]. This method enables unsupervised clustering of tumor tissue, identifying subregions characterized by specific metabolite abundance and revealing intratumoral biological heterogeneity. However, the application of deep learning in proteomics has been relatively slow, mainly due to challenges such as lack of reference standards, accurate analytical methods, computational resources, expertise, and interpretability issues. Since deep learning models require large datasets to be effectively trained, small sample sizes reduce their sensitivity and accuracy in predicting metabolic phenotypes [59], [60], [61]. Despite these challenges, our research group is working on characterizing the proteomic signature of peripheral plasma of influenza patients through deep learning methods. This is particularly obvious in some current studies, especially in small sample clinical studies, and the generalization ability and accuracy of deep learning models are still facing challenges. It is worth noting that compared with previous studies, our research team is trying to solve these challenges by optimizing the deep learning algorithm and combining with rich clinical data. Specifically, we overcome the influence of small sample size on the accuracy of the model by adopting more efficient feature extraction methods and enhanced data enhancement techniques. In addition, our research focuses on the protein omics characteristics of the peripheral plasma of patients with influenza, and explores its potential biomarkers through deep learning methods, so as to provide a new perspective for early diagnosis and precise treatment of influenza.

## Conclusions

We actively searched for differential proteins and identified SAA2 as a potentially key diagnostic clinical marker following influenza infection. In summary, nucleic acid testing offers high sensitivity for pathogen detection but necessitates specialized equipment and extended time for sample collection, nucleic acid extraction, and amplification. The “window period” associated with immune serum methods for diagnosing infectious diseases can also hinder early treatment efforts. Notably, the variations in inflammatory factor production resulting from the immune response to different pathogens may present an effective strategy for the rapid diagnosis of certain infectious diseases. However, our study also has certain limitations, lacking the prevalence and accuracy of these biomarkers to be verified in a larger population. In the future, a multi-center collaboration can be established to conduct follow-up studies. Our study contributes valuable insights for the clinical identification of potential diagnostic markers.

## Abbreviations

Data-dependent acquisition (DDA).

principal component analysis (PCA).

least absolute shrinkage and selection operator (LASSO).

gene ontology (GO).

Kyoto Encyclopedia of Genes and Genomes (KEGG).

weighted gene co-expression network analysis (WGCNA).

topological overlap measure (TOM).

Cell-type Identification by Estimating Relative Subsets of protein (CIBERSORT).

differentially expressed proteins (DEPs).

interleukin-6 (IL-6).

procalcitonin (PCT).

pathogen-associated molecular patterns (PAMPs).

pattern recognition receptors (PRRs).

serum amyloid A (SAA).

Deep learning (DL).

convolutional neural network (CNN).

mass spectrometry (MS).

desorption electrospray ionization (DESI).

## Compliance with Ethics Requirements

This study was reviewed and approved by the Ethics Committee of the Second Affiliated Hospital of Mudanjiang Medical College (ethics number: 202328). Informed consent was obtained from all individual participants in the study. This study involving human research participants was conducted in accordance with the Declaration of Helsinki.

## Authors' contributions

WL and WJ designed the topic and provided key guidance in the writing of the paper. WL and ZYM were responsible for checking the data and writing the paper and were responsible for the revision. ZYM, WPB and WL drew the figures. LS and WL were responsible for collecting the clinical data and blood samples of the patients. ZHY and MX were responsible for separating peripheral blood plasma and ELISA testing. WTH, BMH, FPB and LS collated the data and converted it from paper form to electronic format. All authors read and approved the final manuscript. The corresponding authors are Lei Wang and Ji Wang were responsible for the interpretation of the paper.

## Declaration of competing interest

The authors declare that they have no known competing financial interests or personal relationships that could have appeared to influence the work reported in this paper.
