# Supplementary material for: Prediction of influenza virus infection based on deep learning and peripheral blood proteomics: A diagnostic study
Source: J Adv Res. 2025 Mar 28;79:707–19. doi: 10.1016/j.jare.2025.03.051 (PMC12766177; doi:10.1016/j.jare.2025.03.051)
Supplement: Supplementary Data 1 [file mmc1.docx]

**Supplementary materials**


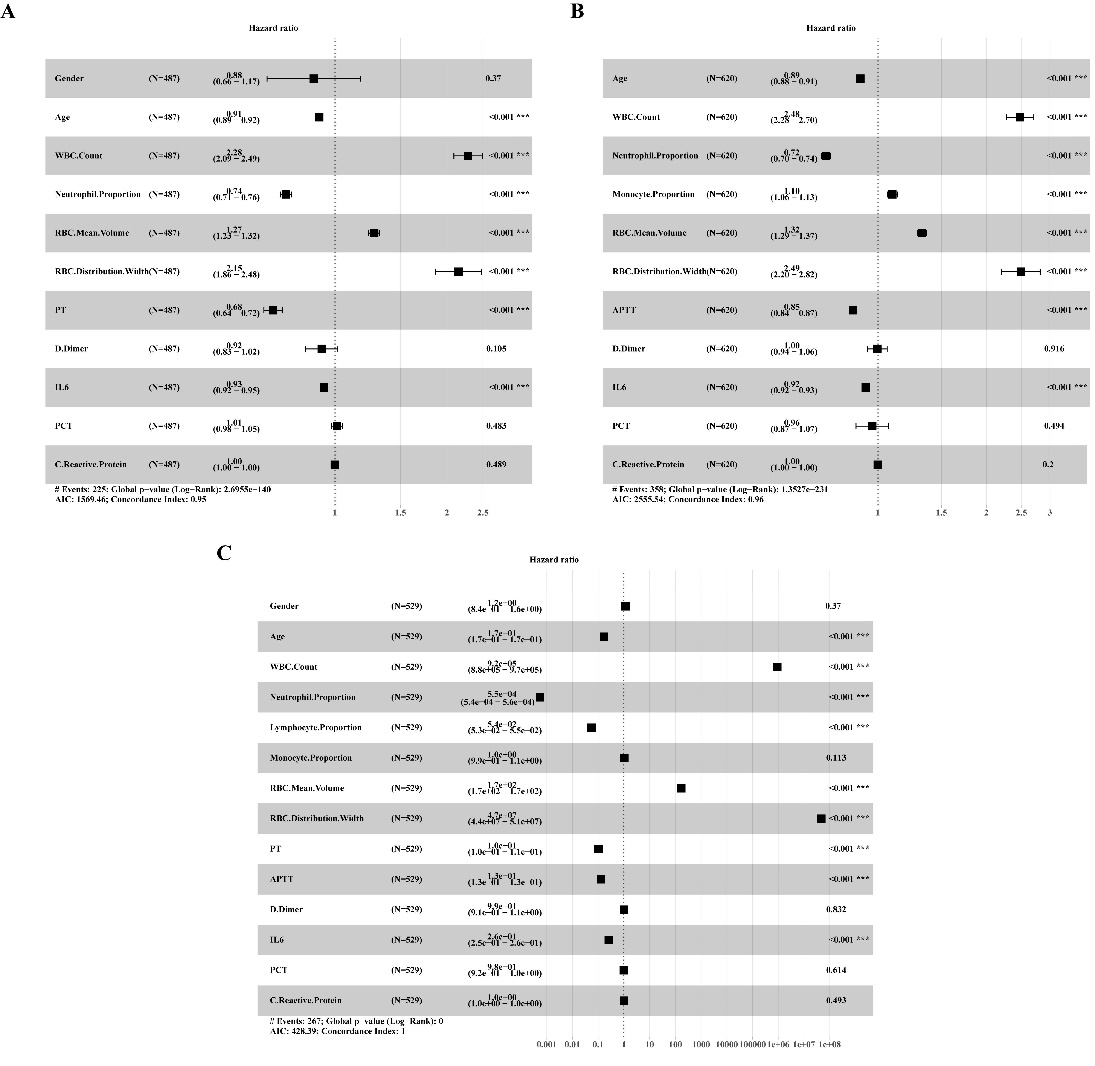


**Fig. S1.** **screen of clinical sample features.** A. Clinical Feature Selection in the Influenza. B. Clinical Feature Selection in the COVID-19. C. Clinical Feature Selection in the Mix.


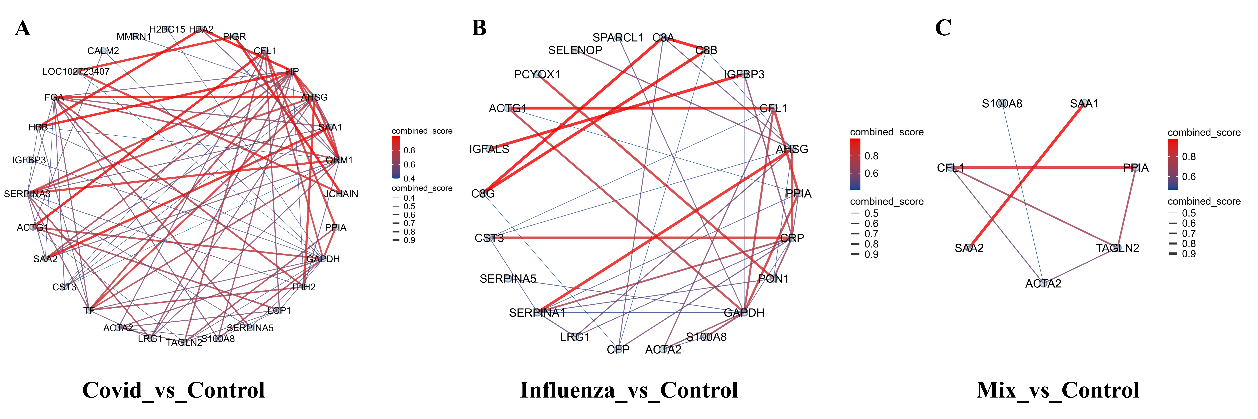


**Fig. S2. differential protein-protein interaction network.**


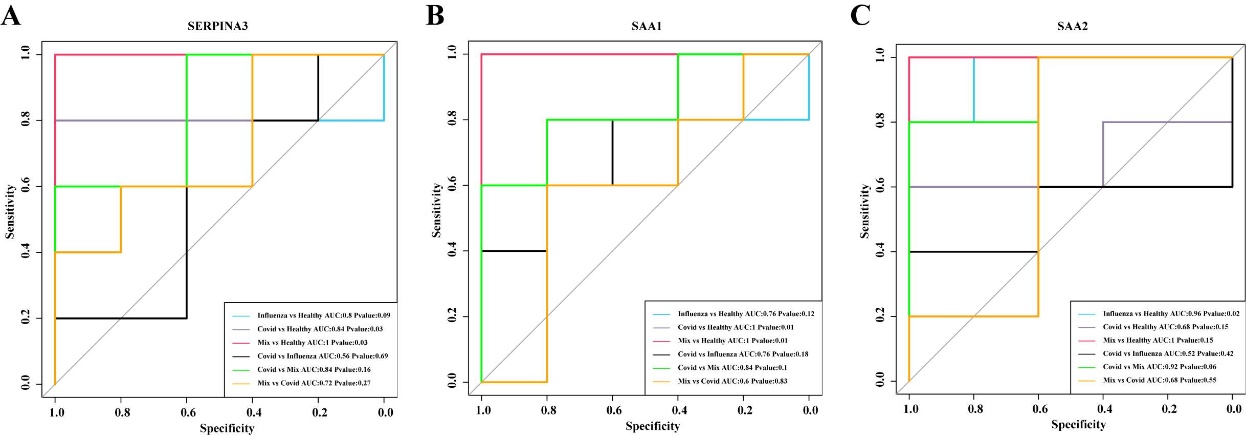


**Fig. S3.** **SERPINA3, SAA1, and SAA2 biomarker identification.** A. ROC curve for **SERPINA3** in proteomic sequencing (Influenza *vs* Healthy, AUC=0.8; Covid *vs* Healthy, AUC=0.84; Mix *vs* Healthy, AUC=1; Covid *vs* Influenza, AUC=0.56; Covid *vs* Mix, AUC=0.84; Mix *vs* Covid, AUC=0.72); B. ROC curve for **SAA1** in proteomic sequencing (Influenza *vs* Healthy, AUC=0.76; Covid *vs* Healthy, AUC=1; Mix *vs* Healthy, AUC=1; Covid *vs* Influenza, AUC=0.76; Covid *vs* Mix, AUC=0.84; Mix *vs* Covid, AUC=0.6); C. ROC curve for **SAA2** in proteomic sequencing (Influenza *vs* Healthy, AUC=0.96; Covid *vs* Healthy, AUC=0.15; Mix *vs* Healthy, AUC=1; Covid *vs* Influenza, AUC=0.52; Covid *vs* Mix, AUC=0.92; Mix *vs* Covid, AUC=0.68).

**Table S1 Clinical data analysis**

|  | **Overall** | **Control** | **Influenza** | **Covid** | **Mix** | ***p*** |
| --- | --- | --- | --- | --- | --- | --- |
| **n** | **1115** | **265** | **225** | **358** | **267** |  |
| **Age (mean (SD))** | **65.14 (16.92)** | **60.45 (19.18)** | **63.21 (18.47)** | **68.77 (14.39)** | **66.54 (14.94)** | **<0.001** |
| **WBC.Count (mean (SD))** | **7.66 (4.20)** | **7.71 (4.47)** | **9.13 (4.32)** | **6.98 (3.39)** | **7.30 (4.50)** | **<0.001** |
| **Neutrophil.Proportion (mean (SD))** | **70.04 (17.04)** | **66.07 (17.38)** | **70.72 (17.19)** | **70.83 (16.05)** | **72.37 (17.30)** | **<0.001** |
| **Lymphocyte.Proportion (mean (SD))** | **17.89 (12.27)** | **21.66 (15.42)** | **17.29 (12.18)** | **17.10 (10.45)** | **15.72 (10.17)** | **<0.001** |
| **Monocyte.Proportion (mean (SD))** | **8.13 (5.48)** | **8.53 (4.49)** | **7.71 (6.54)** | **8.54 (6.21)** | **7.55 (4.14)** | **0.054** |
| **RBC.Mean.Volume (mean (SD))** | **89.17 (13.91)** | **91.30 (8.20)** | **89.22 (14.13)** | **88.79 (14.70)** | **87.51 (16.68)** | **0.016** |
| **RBC.Distribution.Width (mean (SD))** | **13.18 (3.39)** | **14.19 (2.58)** | **13.07 (2.48)** | **12.70 (2.43)** | **12.90 (5.18)** | **<0.001** |
| **PT (mean (SD))** | **9.76 (5.77)** | **8.38 (6.48)** | **9.79 (5.72)** | **10.29 (5.07)** | **10.40 (5.74)** | **<0.001** |
| **APTT (mean (SD))** | **21.41 (12.37)** | **18.36 (13.79)** | **20.81 (11.95)** | **23.26 (11.43)** | **22.48 (11.87)** | **<0.001** |
| **D.Dimer (mean (SD))** | **0.96 (2.01)** | **0.99 (1.86)** | **0.86 (1.69)** | **0.96 (2.30)** | **1.01 (1.98)** | **<0.001** |
| **IL6 (mean (SD))** | **6.89 (22.05)** | **1.14 (5.63)** | **4.91 (17.76)** | **11.25 (31.03)** | **8.43 (19.55)** | **<0.001** |
| **PCT (mean (SD))** | **0.46 (2.72)** | **0.56 (3.97)** | **0.77 (3.28)** | **0.22 (1.13)** | **0.40 (2.10)** | **<0.001** |
| **C.Reactive.Protein (mean (SD))** | **36.82 (57.58)** | **42.41 (52.10)** | **35.47 (53.53)** | **33.68 (53.20)** | **36.62 (70.23)** | **<0.001** |
| **Gender = 2 (%)** | **492 (44.1)** | **119 (44.9)** | **97 (43.1)** | **151 (42.2)** | **125 (46.8)** | **<0.001** |

*p value represents the comparison between Influenza and Control*

**Table S2 LASSO Regression Feature Coefficients**

| **Features** | **Influenza** | **Features** | **Covid** | **Features** | **Mix** |
| --- | --- | --- | --- | --- | --- |
| WBC Count | 0.013 | Neutrophil Proportion | 0.006 | Gender | 0.028 |
| PT | 0.007 | Age | 0.005 | PT | 0.009 |
| IL6 | 0.004 | APTT | 0.005 | Neutrophil Proportion | 0.009 |
| Neutrophil Proportion | 0.002 | Monocyte Proportion | 0.003 | IL6 | 0.005 |
| Age | 0.002 | IL6 | 0.003 | Age | 0.004 |
| PCT | 0.001 | Gender | 0.000 | Monocyte Proportion | 0.003 |
| Lymphocyte Proportion | 0.000 | Lymphocyte Proportion | 0.000 | APTT | 0.001 |
| Monocyte Proportion | 0.000 | PT | 0.000 | Lymphocyte Proportion | 0.000 |
| APTT | 0.000 | C Reactive Protein | -0.001 | C Reactive Protein | -0.001 |
| C Reactive Protein | -0.001 | RBC Mean Volume | -0.001 | PCT | -0.004 |
| Gender | -0.001 | PCT | -0.006 | RBC Mean Volume | -0.009 |
| RBC Mean Volume | -0.003 | D-Dimer | -0.008 | WBC Count | -0.011 |
| D-Dimer | -0.014 | WBC Count | -0.010 | D-Dimer | -0.018 |
| RBC Distribution Width | -0.038 | RBC Distribution Width | -0.054 | RBC Distribution Width | -0.020 |

| **Table S3 Analysis of significant differentially expressed proteins (Covid *vs* Control)** | | | |
| --- | --- | --- | --- |
| Gene Name | Covid vs Control diff | Covid vs Control p.val | Up/Down |
| CRP | 4.90155 | 0.001145 | Up |
| SAA1 | 4.860025 | 0.000915 | Up |
| TAGLN2 | 3.857597 | 0.000583 | Up |
| CFL1 | 3.264524 | 0.002592 | Up |
| PPIA | 3.205434 | 0.000163 | Up |
| LRG1 | 3.192388 | 0.037871 | Up |
| ACTA2 | 3.172272 | 0.001075 | Up |
| SAA2 | 2.662859 | 0.036195 | Up |
| S100A8 | 2.656771 | 0.003296 | Up |
| F5-20 | 2.390755 | 0.034663 | Up |
| DEFA3 | 2.233109 | 0.036021 | Up |
| PIGR | 2.089744 | 0.026861 | Up |
| HEL-S-2 | 2.020658 | 0.009043 | Up |
| GAPDH | 1.978284 | 0.007573 | Up |
| H2BC15 | 1.810834 | 0.016557 | Up |
| ACTG1 | 1.770206 | 0.004245 | Up |
| HBB | 1.632773 | 0.045317 | Up |
| HEL-213 | 1.625745 | 0.011939 | Up |
| HBA2 | 1.593514 | 0.049755 | Up |
| HP | 1.562568 | 0.013818 | Up |
| HEL-S-37 | 1.420851 | 0.008994 | Up |
| CALM2 | 1.357783 | 0.031804 | Up |
| FGA | 1.087182 | 0.02679 | Up |
| SERPINA3 | 0.948996 | 0.020297 | Up |
| HEL-S-153w | 0.880425 | 0.023546 | Up |
| TF | -0.74799 | 0.043579 | Down |
| ITIH2 | -0.84462 | 0.02035 | Down |
| BTD | -0.85453 | 0.008712 | Down |
| JCHAIN | -0.92689 | 0.035691 | Down |
| PGLYRP2 | -1.07707 | 0.007685 | Down |
| AHSG | -1.16882 | 0.012632 | Down |
| MMRN1 | -1.20667 | 0.045119 | Down |
| IGFBP3 | -1.4458 | 0.03411 | Down |
| IGH | -1.71097 | 0.04751 | Down |
| CRTAC1 | -2.17707 | 0.006653 | Down |
| SERPINA5 | -2.46155 | 0.002193 | Down |

| **Table S4 Analysis of significant differentially expressed proteins (Influenza *vs* Control)** | | | |
| --- | --- | --- | --- |
| Gene Name | Influenza vs Control diff | Influenza vs Control p.val | Up/Down |
| TAGLN2 | 3.664948 | 0.000935 | Up |
| CRP | 3.571436 | 0.01187 | Up |
| LRG1 | 3.41421 | 0.027525 | Up |
| CFL1 | 2.852642 | 0.006948 | Up |
| S100A8 | 2.501936 | 0.005128 | Up |
| PPIA | 2.424169 | 0.002222 | Up |
| ACTA2 | 2.123071 | 0.018611 | Up |
| ACTG1 | 1.521309 | 0.011714 | Up |
| HEL-S-2 | 1.51437 | 0.042334 | Up |
| GAPDH | 1.438798 | 0.042856 | Up |
| HEL-213 | 1.326284 | 0.035302 | Up |
| SERPINA1 | 0.917846 | 0.039741 | Up |
| BTD | -0.6636 | 0.035132 | Down |
| CFP | -0.67964 | 0.040147 | Down |
| C8G | -0.72861 | 0.032833 | Down |
| SELENOP | -0.81685 | 0.040462 | Down |
| PCYOX1 | -0.84326 | 0.038504 | Down |
| C8B | -0.84468 | 0.02435 | Down |
| C8A | -0.87132 | 0.011486 | Down |
| PGLYRP2 | -0.91873 | 0.019938 | Down |
| PON1 | -0.98863 | 0.029068 | Down |
| IGFALS | -1.18234 | 0.012639 | Down |
| AHSG | -1.18829 | 0.011429 | Down |
| SPARCL1 | -1.61811 | 0.042894 | Down |
| IGFBP3 | -1.94934 | 0.006145 | Down |
| SERPINA5 | -2.63923 | 0.001222 | Down |

| **Table S5 Analysis of significant differentially expressed proteins (Mix *vs* Control)** | | | |
| --- | --- | --- | --- |
| Gene Name | Mix vs Control diff | Mix vs Control p.val | Up/Down |
| CRP | 5.307905 | 0.000552 | Up |
| TAGLN2 | 5.25372 | 2.01E-05 | Up |
| SAA1 | 5.18693 | 0.000499 | Up |
| CFL1 | 4.169258 | 0.000286 | Up |
| SAA2 | 3.789832 | 0.00459 | Up |
| PPIA | 3.771765 | 2.56E-05 | Up |
| ACTA2 | 3.402453 | 0.000565 | Up |
| S100A8 | 2.931093 | 0.001493 | Up |
| IGLC7 | -3.06354 | 0.008413 | Down |
| IGFBP3 | -3.54209 | 2.03E-05 | Down |
